# Standardized Ultrasound Protocol for Peripherally Inserted Central Catheters in Neonates: A Retrospective, X-ray Controlled Observational Study

**DOI:** 10.3390/children11101204

**Published:** 2024-09-30

**Authors:** Oliver Firszt, Magdalena Maślanka, Agata Grabowska, Ewa Kluczewska

**Affiliations:** 1 Doctoral Studies School, Medical University of Silesia, 40-055 Katowice, Poland; 2 Department of Radiology in Zabrze, Medical University of Silesia, 40-055 Katowice, Poland; roenzab@sum.edu.pl; 3 Neonatal Pathology Unit, Provincial Hospital in Bielsko-Biała, 43-316 Bielsko-Biala, Poland

**Keywords:** neonatal intensive care unit, PICC, ultrasound, neonate

## Abstract

**Objectives:** Ultrasound (US) has been reported to be a reliable imaging modality for locating the tip of peripherally inserted central venous catheters (PICC) in neonates. However, its use requires a certain level of expertise, which may limit its application. Standardization of US examinations using designed protocols may improve their efficiency and ease of use. The objective of our study is to evaluate the effectiveness of introducing a standardized US protocol for PICC guidance. **Methods:** An expert panel was formed in order to develop a standardized US protocol for PICC assessment. Institutional review board agreement was obtained. This protocol was then used by participating clinicians to assess PICC position. Every assessment was followed by a radiographic control. The study group consisted of 262 US examinations of PICC lines in a level III neonatal intensive care unit (NICU) performed according to the designed protocol. Subsequent statistical analysis was made with the RStudio software, R version 4.3.1 (2023-06-16 ucrt). **Results:** Compared to the X-ray control, standardized US examinations showed 100% sensitivity, 81% specificity, an accuracy of 98% and a 98% precision for assessing catheter placement. The Cohen’s Kappa value for the comparison of X-ray and US studies was 0.88, indicating good agreement between the two methods. **Conclusions:** Standardized US examinations have shown similar potential for evaluating the location of PICC lines when compared with radiographic studies. Implementing a standardized protocol in the NICU may facilitate the ultrasound assessment of PICC lines and reduce exposure to ionizing radiation.

## 1. Introduction

PICC venous catheters are an important and widely used type of vascular access in the NICU [1]. They are largely reserved for short and medium-term continuous infusions, especially parenteral nutrition. Other drugs, such as highly osmolar solutions or drugs with a pH of <5 or >9, are also administered through PICC lines. Nutrition is one of the key elements in the care of preterm infants, especially for very low birth weight (VLBW) and extremely low birth weight (ELBW) newborns [2]. Therefore, this type of vascular access is most commonly used in less mature and smaller neonates. Despite the advances in US implementation, NICU patients still tend to undergo a significant number of X-ray examinations, with the smallest and most premature neonates being the most at risk [3]. It is necessary to verify the correct placement of a central catheter in order to minimise potential complications [4]. Classic radiography is still the main imaging modality for this task in many NICUs, which adds to the burden of patient X-ray exposure [1,5].

Ultrasound has been successfully used as an imaging modality to guide PICC placement in both adult patients and neonates [5,6,7]. Attempts to establish a more standardised, ultrasound-based approach to PICC insertion in adults date back to the late 1990s [7]. Similar efforts in neonatology remain scarce, and scientific, outcome-based data lacking. A recent study by Barone et al. is a notable example of such an attempt, proposing a simple protocol for central catheter imaging [5]. However, it is an editorial article, and the proposed protocol is based on a literature search and the authors’ experience. To our best knowledge, there are no studies evaluating the development and implementation of an internal protocol for PICC tip location in the NICU. Performing US studies according to designed protocols, developing guidelines and imaging standards has been reported to improve the quality of US studies and optimise learning curves [8]. While this is also true for point-of-care ultrasound (POCUS), learning curves vary depending on the protocol and the areas scanned [9]. In this context, neonatal POCUS presents its own challenges, with the relative scarcity of training curricula and the lack of outcome-based guidelines and standardised protocols being important barriers to more widespread adoption in many NICUs [10,11]. Evaluating PICC lines using ultrasound in neonates poses its own challenges, since it requires the visualization of various peripheral and central venous vessels [5]. Our clinical staff has a long history of regular point-of-care ultrasound use, with all the participants having finished dedicated point-of-care (POCUS) courses. However, despite their experience in US and having access to a US machine at all times, most still opted for radiographic studies to evaluate PICC placement. We put forth a hypothesis that creating a standardized protocol could facilitate and encourage ultrasound PICC assessment in our unit. This idea was grounded in our experiences in introducing a lung ultrasound assessment protocol, which markedly reduced chest X-ray use in our unit.

The aim of our work was to evaluate an expert-developed PICC placement assessment protocol and its implementation in a neonatal intensive care unit. To ensure patient safety, as requested by an institutional review board, the ultrasound examinations were compared with radiographic studies. Our results demonstrate the potential of internally developed protocols to facilitate US performance.

## 2. Materials and Methods

The work was planned as an observational cohort study in a group of neonates who required PICC insertion during their hospital stay, and subsequently underwent both a standardized, protocol-guided ultrasound assessment and an X-ray study for PICC assessment. The observations were made in a level III neonatal unit in Bielsko-Biała, Poland. The project was approved by an institutional review board and the Bioethics Committee of the Medical University of Silesia, decision no. BNW/NWN/0052/KB/16/24. The study group consisted of 262 US examinations performed according to a standardised protocol in 177 neonates, each followed by an X-ray study. Inclusion criteria for the study required the examination to be documented, and the radiograph to be externally reviewed by a radiologist. Participants documented their ultrasound findings using either still images or short clips showing the tip of the catheter. Catheter positioning was described as correct, incorrect or uncertain in the imaging studies. The place of insertion, catheter’s length, body weight and body length were among the evaluated variables. Optimal PICC positions used in our unit are described in the study protocol found in Supplementary Materials. The characteristics of the study cohort are shown in Table 1.

Statistical analysis was performed using RStudio software. The normality of the distribution of variables was assessed using a generated histogram and quantile-quantile (qq) plots, both generated using RStudio. Relationships between variables were assessed using chi-squared tests for qualitative variables. T-student test was used for quantitative variables with a near-normal distribution and Wilcoxon test for those with a non-normal distribution, with a significance level of *p* < 0.05. Cohen’s kappa was used to assess agreement between studies.

The ultrasound protocol for PICC assessment was developed by a working group of experienced clinicians, including neonatologists, radiologists and anaesthetists. Over a series of meetings, a literature review identified existing scientific data, which was analysed and discussed. This was followed by a series of hands-on sessions and discussions with NICU clinicians to identify and address unit-specific issues and needs, including the creation of imaging presets for the available US machines. The findings of the working group were then incorporated into a concise examination protocol, which included suggested views that were designed to be easily reproducible, using standard linear and curvilinear transducers. The final study protocol is presented in Supplementary Materials and is intended to be used either after placement of a PICC line or to guide its placement in real time.

The first step was to identify the vessel as a vein using a linear high frequency probe with a superficial preset. In ultrasound images, veins are characterised by their susceptibility to compression, with the more rigid walls of arteries requiring a greater force to compress them. In addition, the arterial pulse sign is easily visible under slight compression. When no force is applied, venous vessels may also pulsate if they are close to an artery, and the wall thickness of the smallest vessels is difficult to assess, thus making it difficult to differentiate between veins and arteries based on pulse sign alone.

### 2.1. Lower Limb Access

**Figure 1:**
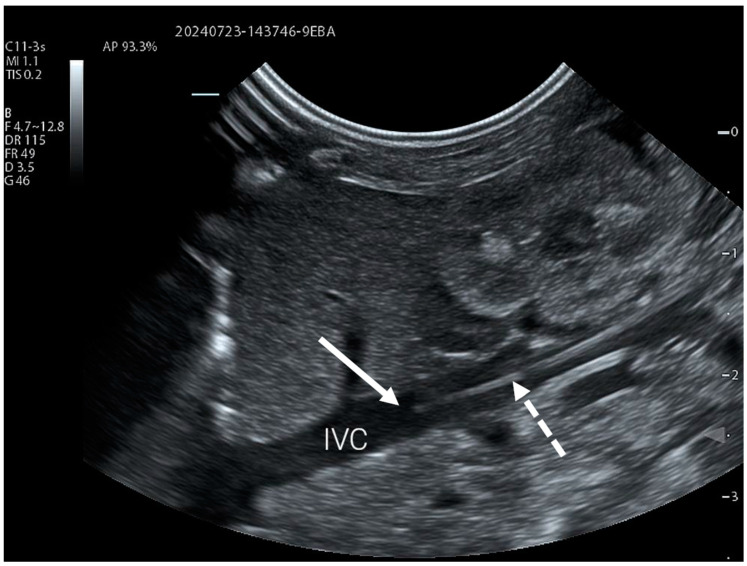
PICC catheter clearly visible in the inferior vena cava (IVC) using a simple lateral abdominal view. The catheter is marked with a dotted arrow, and the full arrow indicates its tip.

For PICC lines inserted from the lower limb, a lateral abdominal view of the right flank is used to visualise the IVC using either a curvilinear or linear transducer. This acoustic window is not only easy to use, but also allows visualisation of tributary veins to the IVC, such as the renal veins (Figure 1, Video S1). The right abdominal flank is more suitable for such a view, not only because of the anatomy of the vena cava, but also to avoid the artefacts caused by gastric contents on the left side.

If the catheter was not visualised within the IVC, a transverse view of the vessels in the groin area was taken to visualise the common femoral artery (CFA), common femoral vein (CFV) and sapheno-femoral junction. Failure to visualise the catheter within these vessels was considered an indication of misplacement or operator error (i.e., choosing a PICC line that’s too short).

### 2.2. Upper Limb Access

**Figure 2:**
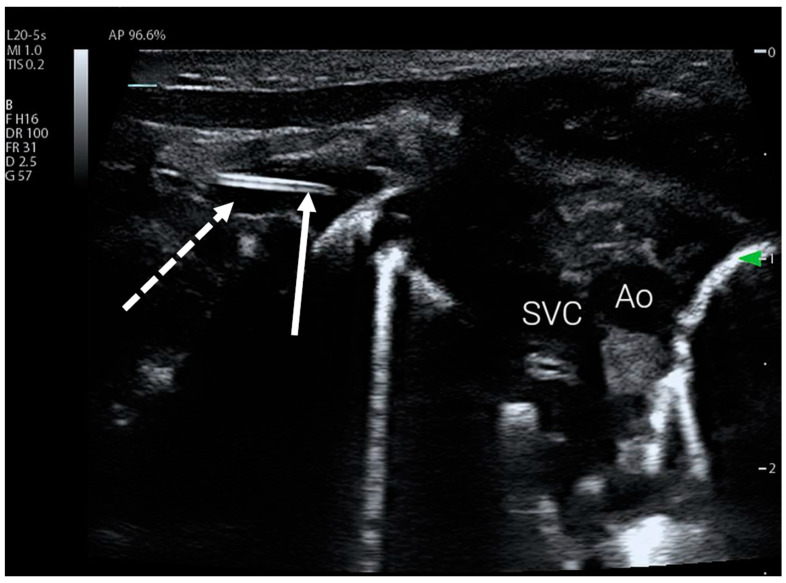
PICC catheter tip visible in the subclavian vein using subclavian view. The subclavian vein is marked with a dotted arrow, full arrow indicates catheter tip. Ao—aorta, SVC—superior vena cava.

For upper limb PICC access, the protocol uses a subclavicular view to visualise the mediastinum and the subclavian vein (Figure 2). This acoustic window is also easy to obtain and has the advantage of visualising the venous junction between the subclavian vein and the superior vena cava (SVC), which is the innominate vein. If the catheter was not visualised, a view through the axillary fossa was obtained using either a linear or curvilinear transducer to visualise the axillary vein and the distal part of the subclavian vein. Failure to visualise the catheter using this view was considered an indication of malposition or inadequate catheter length. If the catheter was visible within the subclavian vein but its tip was difficult to visualise, the protocol required the user to obtain a simple transverse view of the jugular vessels and the contralateral subclavian vein to rule out tip position in these vessels.

### 2.3. Ultrasound Presets

For ease of use and to remove the burden of setting the imaging parameters from the clinician, 4 simple presets were created—2 for a curvilinear (convex) probe and 2 for a linear probe, either for superficial or deeper imaging. Participants were free to use either, and there were no instances where the settings needed to be changed during our observations.

## 3. Results

262 examinations in 177 newborns were included in the study. The summary results of both ultrasound and X-ray examinations in the study cohort are presented in Table 2.

In relation to radiographic findings, standardised ultrasound examinations using the designed protocol showed 100% sensitivity, 81% specificity, 98% accuracy and 98% precision. The calculated Cohen’s kappa value for comparing US and radiographic results was 0.88, indicating good agreement between the two methods, with an agreement percentage of 98.2%. The results of observed variables for X-ray studies are presented in Table 3.

The results for ultrasound studies are presented in Table 4. Of note, incorrect PICC placement was more often observed in larger and older neonates. Interestingly, incorrect catheter tip localisation was observed more commonly with upper limb access.

Only in 4 cases were the ultrasound results falsely positive for correct PICC placement. In each of these instances, radiographs showed either an unacceptable kink or looping of the catheter. The remaining cases were correctly assessed using ultrasound as the imaging modality. Uninterpretable results were more common in radiographic studies (10%) compared to ultrasound (7.6%).

## 4. Discussion

The concept of using ultrasound to image central venous catheters is not new and is well established in the medical literature in adult patients. In addition, a growing body of evidence supports its use in neonatology [5,10,11]. Our observations remain in agreement with existing data, confirming the method’s viability in evaluating PICC catheters. However, there is some disparity between the actual and potential use of this imaging modality for procedural guidance and placement control of PICC lines. A lack of outcome-based guidelines or adequate training programmes is often cited as a major barrier to more widespread use of ultrasound [11,12]. Similar remarks were voiced by our clinicians, with the need for a reference standard and the uncertainty of the results obtained being identified as major barriers to the adoption of ultrasound as the basic imaging modality for PICC assessment in the unit. The goal of our study was to explore the idea of introducing an internal protocol to facilitate and guide US assessments of PICC lines, and the results obtained indicate this may be feasible in the NICU. Performing US examinations according to a standardised protocol has been shown to affect the learning curves [9]. Therefore, we concluded that the creation of a simple, easy-to-use standardised protocol could facilitate and incentivise the use of ultrasound for this task, leading to a reduction in radiographic studies in the future. Although the available scientific data fully supported the use of ultrasound for this task, our bioethics committee and institutional review board expressed concern about the sudden introduction of a new standard, citing a long history of reliance on classic radiography as the first-line imaging modality. Therefore, it was suggested that initially both be performed, with the aim of comparing the results. Based on our findings, we have confirmed that standardising the ultrasound assessment of PICC lines could facilitate its use and introduction in the NICU. Creating specific examination presets removes the burden of image adjustment from the clinician and speeds up the setup. Furthermore, standardizing acoustic windows allows for easy reproducibility of ultrasound studies. When compared to X-ray studies, as exemplified in our study and available literature, ultrasound retains adequate effectiveness in assessing PICC lines placement. The novelty of our approach is to explore means of introducing the modality to a unit’s clinicians using an examination protocol specifically designed for the task.

It is important to note that our study has several limitations. It is a retrospective, single-centre, observational study with an X-ray control. The clinicians who performed the US scans knew that an x-ray study would follow, so there may be some bias in our assessment of clinicians’ willingness to use the protocol.

## 5. Conclusions

Standardised US examinations performed using the designed protocol have displayed similar effectiveness in assessing the position of PICC lines compared with radiographic examinations. The implementation of a standardised protocol in the NICU may facilitate and encourage ultrasound assessment of PICC lines, potentially reducing exposure to ionising radiation.

## Figures and Tables

**Table 1:** Study cohort characteristics.

Variable	*n* = 262 ^1^	95% CI ^2^
Gestational age at birth (weeks)	30.00 (28.00, 34.00)	30, 32
Body weight (g)	1297.50 (980.00, 1785.00)	1413, 1627
Body length (cm)	41.00 (35.00–46.00)	40, 42
Upper limb access (n, %)	152 (58%)	52%, 64%
Lower limb access (n,%)	109 (42%)	36%, 48%
Other access site (n,%)	1 (0.4%)	0.02%, 2.4%
Side of the body (n,%):		
Left	118 (45%)	39%, 51%
Right	144 (55%)	49%, 61%
Catheter length (n,%):		
10 cm	103 (39%)	33%, 46%
15 cm	120 (46%)	40%, 52%
20 cm	39 (15%)	11%, 20%

^1^ n (%); Median (Q1, Q3) ^2^ CI = Confidence Interval.

**Table 2:** Comparison of US and X-ray PICC assessments.

Variable	*n* = 262 ^1^	95% CI ^2^
**X-ray PICC control (n,%):**		
Incorrect placement	26 (9.9%)	74%, 84%
Correct placement	209 (80%)	7.0%, 15%
Uncertain	27 (10%)	6.7%, 14%
**Ultrasound PICC control (n,%):**		
Incorrect placement	29 (11%)	76%, 86%
Correct placement	213 (81%)	4.8%, 12%
Uncertain	20 (7.6%)	7.7%, 16%

^1^ n (%); Median (Q1, Q3) ^2^ CI = Confidence Interval.

**Table 3:** Comparison of examined variables for X-ray studies.

Variable (X-ray Studies)	Incorrect PICC Placement ^1^ *n* = 26	Correct PICC Placement ^1^ *n* = 209	*p*-Value ^2^
Gestational age (weeks)	28.00 (27.00, 32.00)	30.00 (28.00, 33.00)	0.12
Body weight (g)	990.00 (870.00, 1300.00)	1300.00 (980.00, 1760.00)	0.048 *
Body length (cm)	38.50 (34.00, 42.00)	41.00 (35.00, 45.00)	0.23
Localisation:			0.11
Upper limb access (n, %)	20 (77%)	116 (56%)	
Lower limb access (n, %)	6 (23%)	92 (44%)	
Other	0 (0%)	1 (0.5%)	
Side of the body (n, %):			0.61
Left	13 (50%)	89 (43%)	
Right	13 (50%)	120 (57%)	
Catheter length (n, %):			0.33
10 cm	14 (54%)	81 (39%)	
15 cm	9 (35%)	95 (45%)	
20 cm	3 (12%)	33 (16%)	
Ultrasound assessment			<0.001
Incorrect	17 (65%)	0 (0%)	
Correct	4 (15%)	200 (96%)	
Uncertain	5 (19%)	9 (4.3%)	

^1^ n (%); Median (Q1, Q3). ^2^ Pearson’s Chi-squared test; Welch Two Sample *t*-test. * Wilcoxon’s sum rank test.

**Table 4:** Comparison of examined variables for ultrasound studies.

Variable (Ultrasound Studies)	Incorrect PICC Placement ^1^ *n* = 29	Correct PICC Placement ^1^ *n* = 213	*p*-Value ^2^
Gestational age (weeks)	28.00 (27.00, 32.00)	30.00 (28.00, 33.00)	0.68
Body weight (g)	1275.00 (950.00, 1945.00)	1280.00 (980.00, 1720.00)	0.961 *
Body length (cm)	40.00 (37.00, 47.00)	41.00 (35.00, 45.00)	0.91
Localisation:			0.021
Upper limb access (n, %)	25 (86%)	116 (56%)	
Lower limb access (n, %)	4 (14%)	85 (40%)	
Other	0 (0%)	1 (0.5%)	
Side of the body (n, %):			0.61
Left	16 (55%)	93 (44%)	
Right	13 (45%)	120 (56%)	
Catheter length (n, %):			0.33
10 cm	16 (55%)	86 (40%)	
15 cm	11 (38%)	100 (47%)	
20 cm	2 (6.9%)	27 (13%)	
Ultrasound assessment			<0.001
Incorrect	17 (65%)	0 (0%)	
Correct	4 (15%)	200 (96%)	
Uncertain	5 (19%)	9 (4.3%)	

^1^ n (%); Median (Q1, Q3). ^2^ Pearson’s Chi-squared test; Welch Two Sample *t*-test. * Wilcoxon’s sum rank test.

## Data Availability

All of the data in the study is available upon reasonable request by contacting the corresponding author, including anonymized ultrasound images and data sets.

## Supplementary Materials

The following supporting information can be downloaded at: https://www.mdpi.com/article/10.3390/children11101204/s1, Supplemental File S1: Examination protocol in PDF form; Video S1: A sample video showing catheter placement in the inferior vena cava.
