# Supplementary material for: Standardized Ultrasound Protocol for Peripherally Inserted Central Catheters in Neonates: A Retrospective, X-ray Controlled Observational Study
Source: Children (Basel). 2024 Sep 30;11(10):1204. doi: 10.3390/children11101204 (PMC11505741; doi:10.3390/children11101204)
Supplement: Supplementary file 1 [file children-11-01204-s001.zip › Appendix 1.pdf]

Department of neonatology, Provincial Hospital of Bielsko-Biała, Poland

PICC ULTRASOUND GUIDANCE REFERENCE PROTOCOL

To be used by trained personnel only. This document is intended for internal use in the unit.

Upper limb access

- Use full aseptic technique, including transducer covers for intraprocedural use.
- Always measure estimated PICC length before the procedure.
- Optimal positioning is as close to the right atrium as possible. Proximal part of the subclavian vein, innominate vein and superior vena cava are preferred.
- Suboptimal positioning: distal part of subclavian vein, proximal part of axillary vein. Monitor closely and discuss with senior staff. Please note suboptimal positioning in the PICC monitoring sheet.

Imaging window

- Obtain subclavian view using linear transducer
- Preset name: subclavian PICC
- The catheter tip must be clearly visible within the subclavian vein, innominate vein or superior vena cava.
- Always check jugular vessels for potential tip malposition in the jugular veins on the same side.

Catheter tip not visualized

- Obtain axillary fossa view using curvilinear or linear transducer
- Preset name: microconvex PICC, subclavian PICC
- If the catheter is visible in the axillary vein, evaluate PICC patency and discuss with senior staff
- Proceed with X-ray study if needed

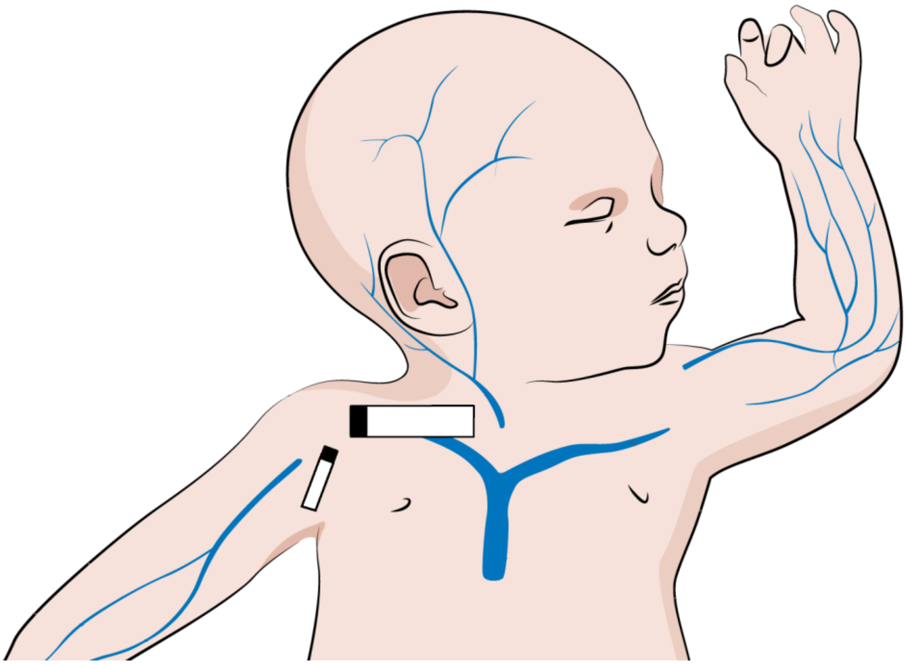

Lower limb access

- Use full aseptic technique, including transducer covers for intraprocedural use.
- Always measure estimated PICC length before the procedure.
- Optimal positioning: inferior vena cava, close to the atrium
- Suboptimal: common iliac vein, internal iliac vein, external iliac vein. Monitor closely and discuss with senior staff. Please note suboptimal positioning in the PICC monitoring sheet.
- Avoid positioning the catheter's tip near renal and lumbar veins. If in doubt, discuss with senior staff.

Imaging window

- Obtain lateral abdominal view using either linear or curvilinear transducer on the right flank.
- Preset name: abdominal PICC (on both linear and curvilinear transducer)
- The catheter tip must be clearly visible within the inferior vena cava or proximal iliac veins.

Catheter tip not visualized

- Obtain inguinal, transverse view using curvilinear or linear transducer
- Preset name: abdominal PICC (on both linear and curvilinear transducer)
- If the catheter is visible in the iliac veins, evaluate PICC patency and discuss with senior staff
- Proceed with X-ray study if needed

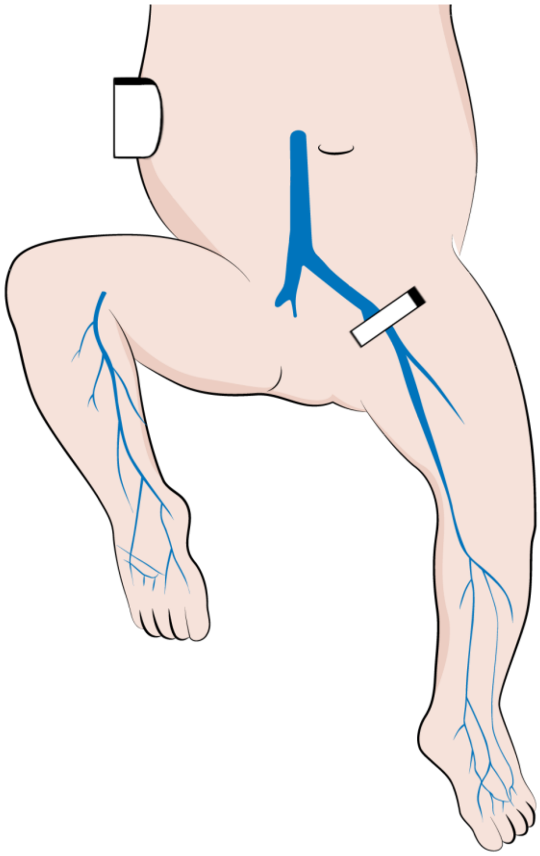

Appendix: reference views

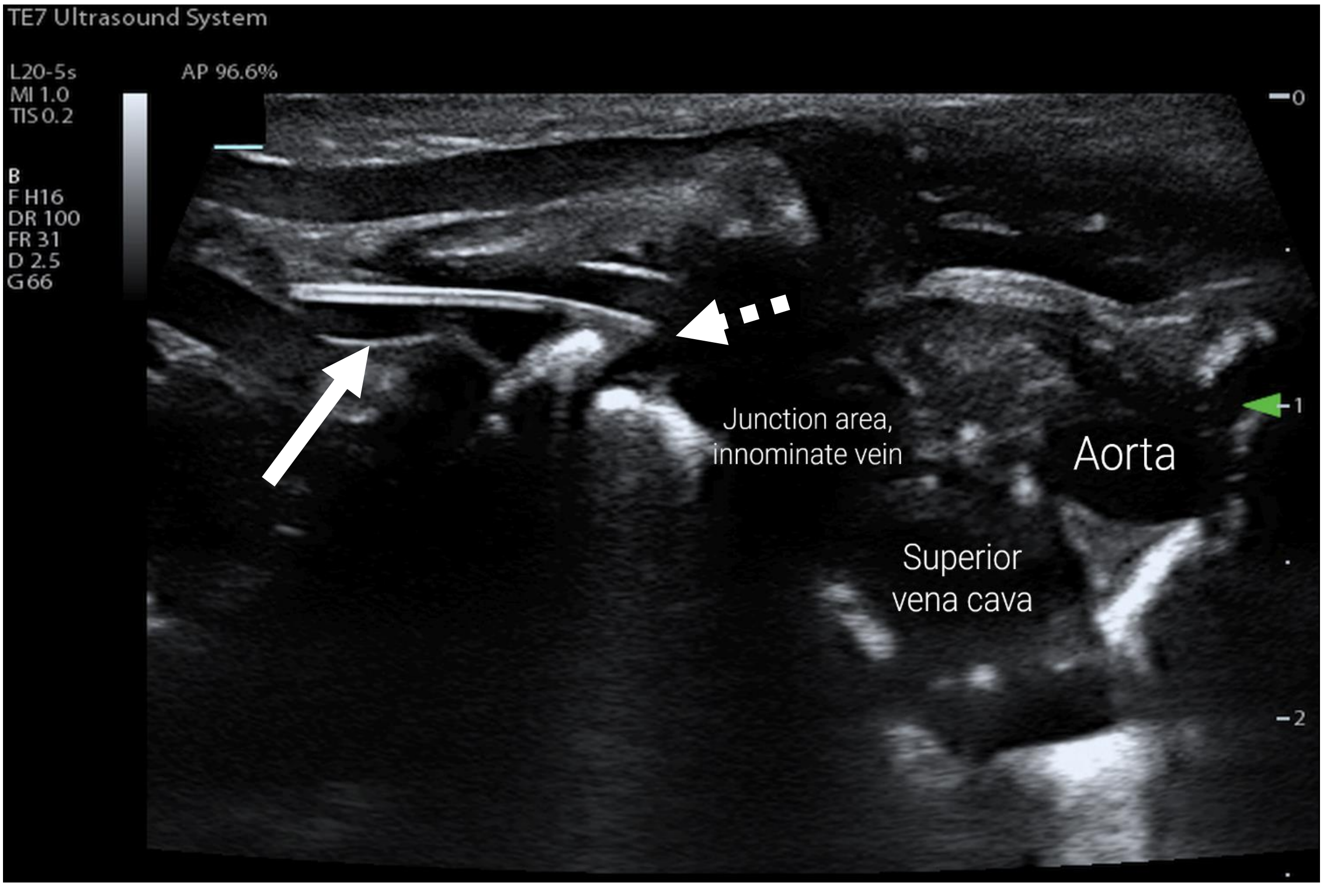

Fig.1: **subclavian view.** Full arrow shows the subclavian vein. Dotted arrow shows PICC tip.

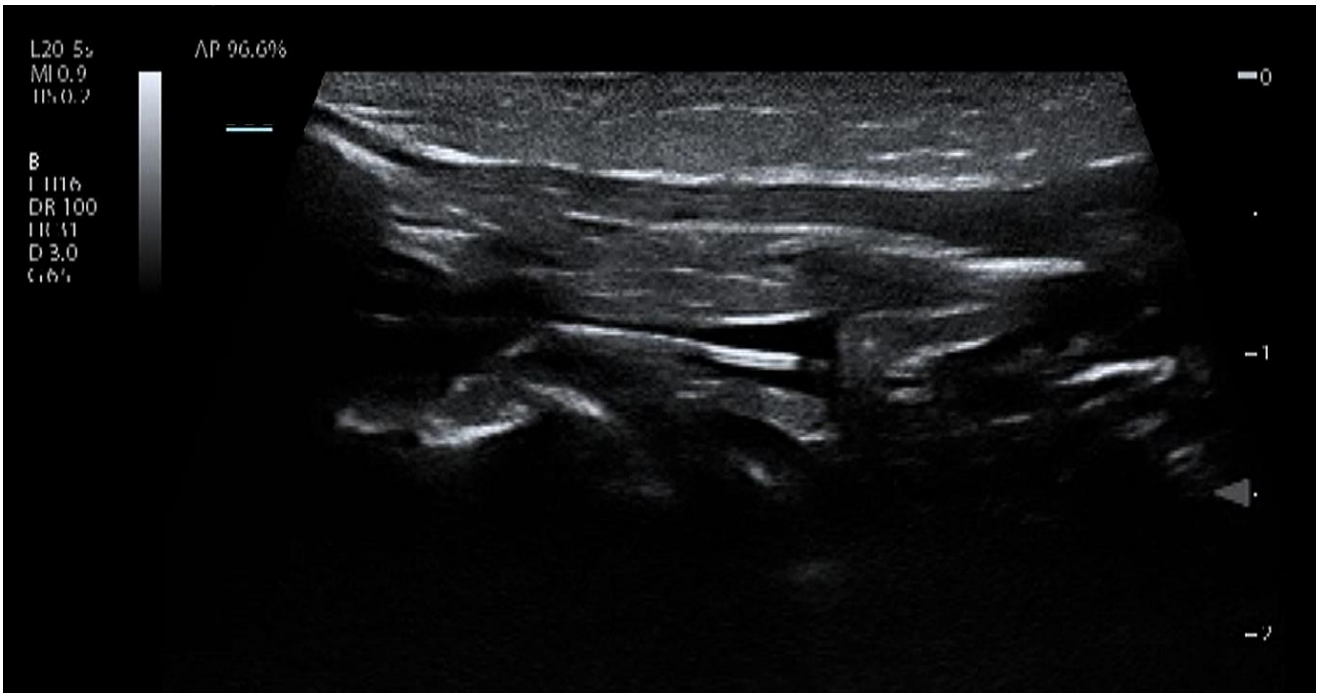

Fig. 2: PICC tip located in the distal segment of the subclavian vein is clearly visible.

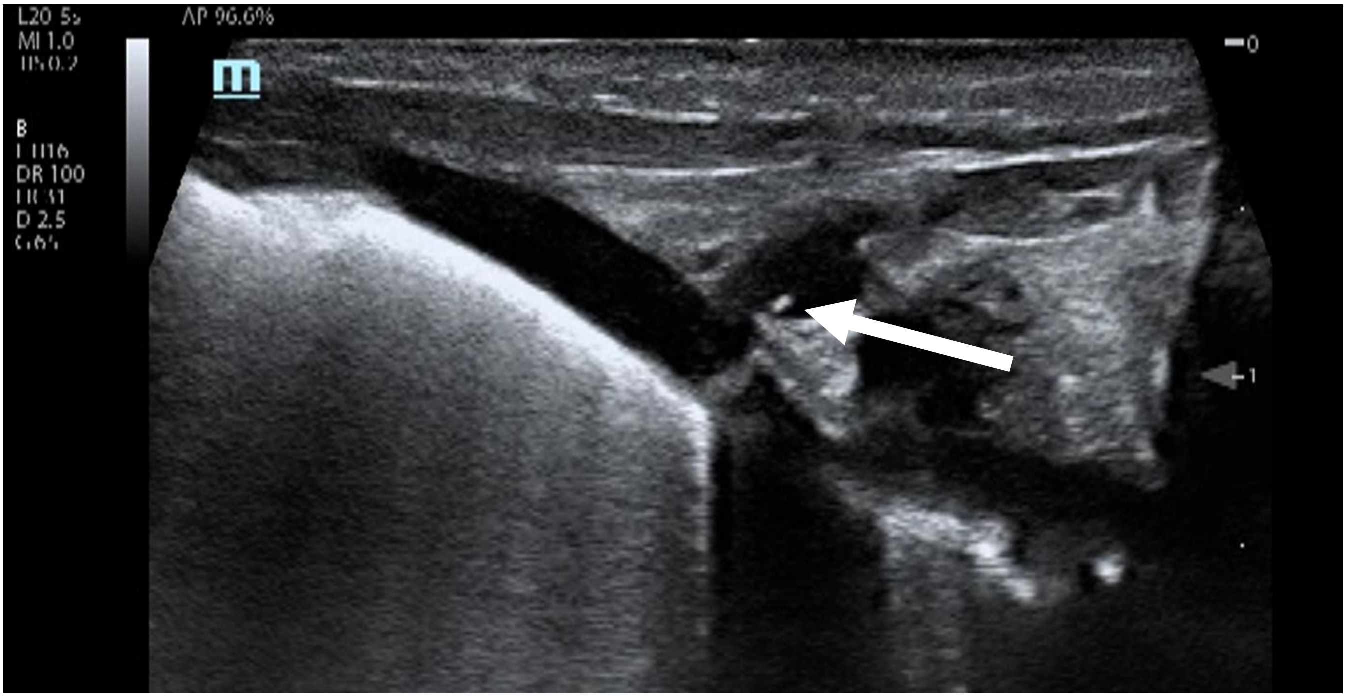

Fig 3. axillary fossa view using linear transducer. Full arrow shows a transverse view of the PICC catheter (white dot) visible in the junction between the axillary vein and subclavian vein.

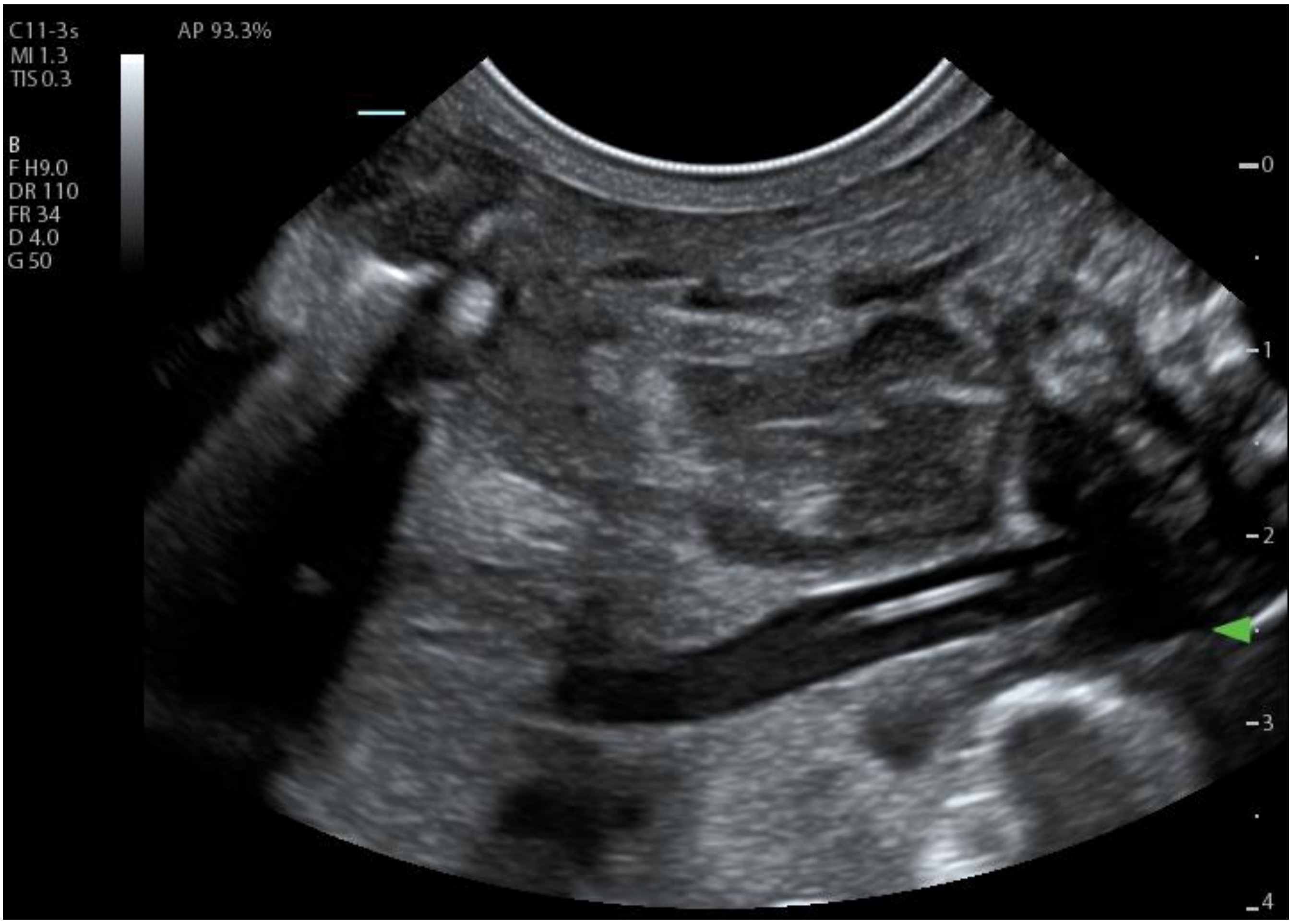

Fig. 4. PICC tip located in the inferior vena cava, above the kidneys, lateral abdominal view. The image of the tip is distorted by the flow of i.v. fluids.

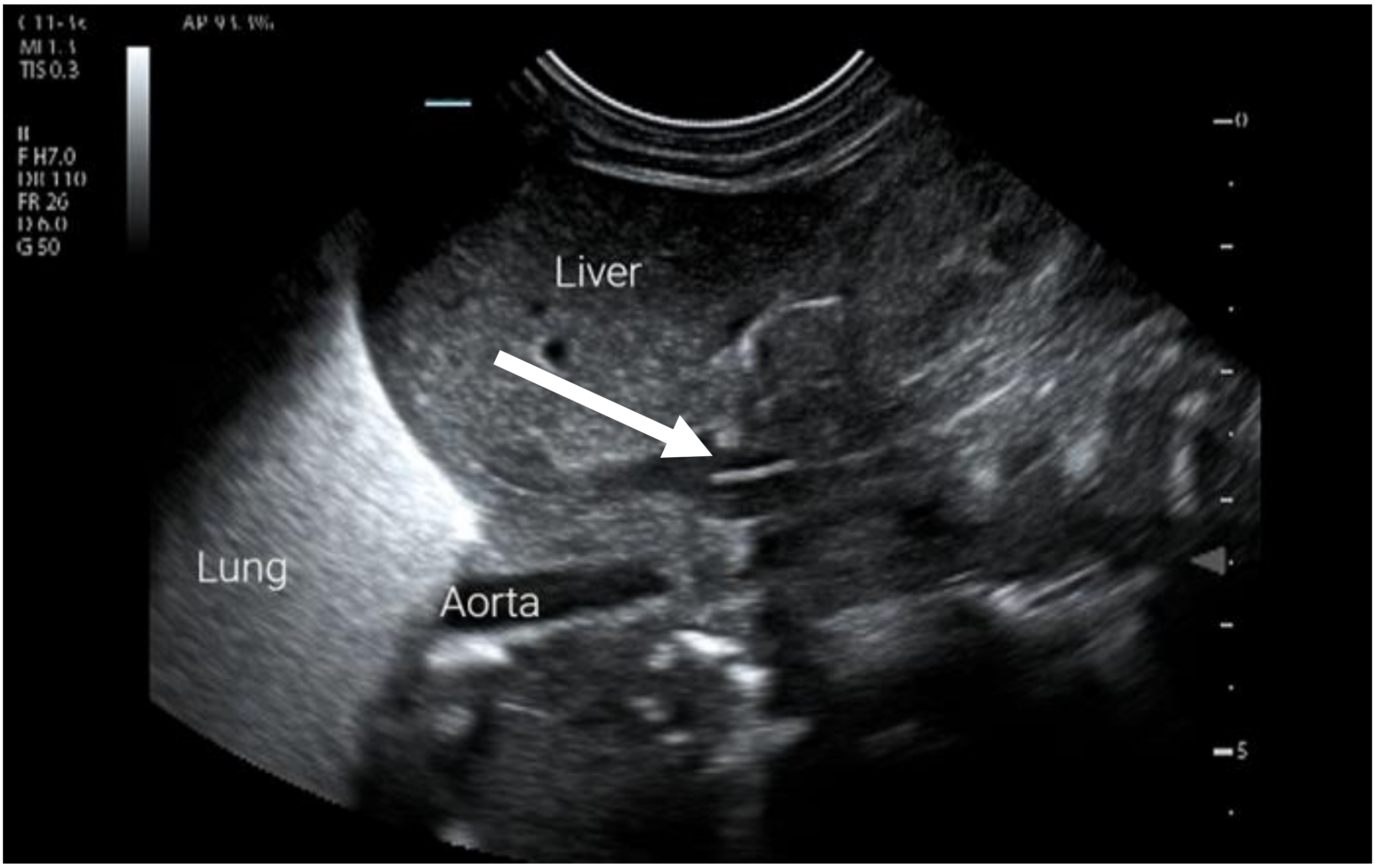

Fig.5. lateral abdominal view. Catheter tip is visible within the inferior vena cava (full arrow).

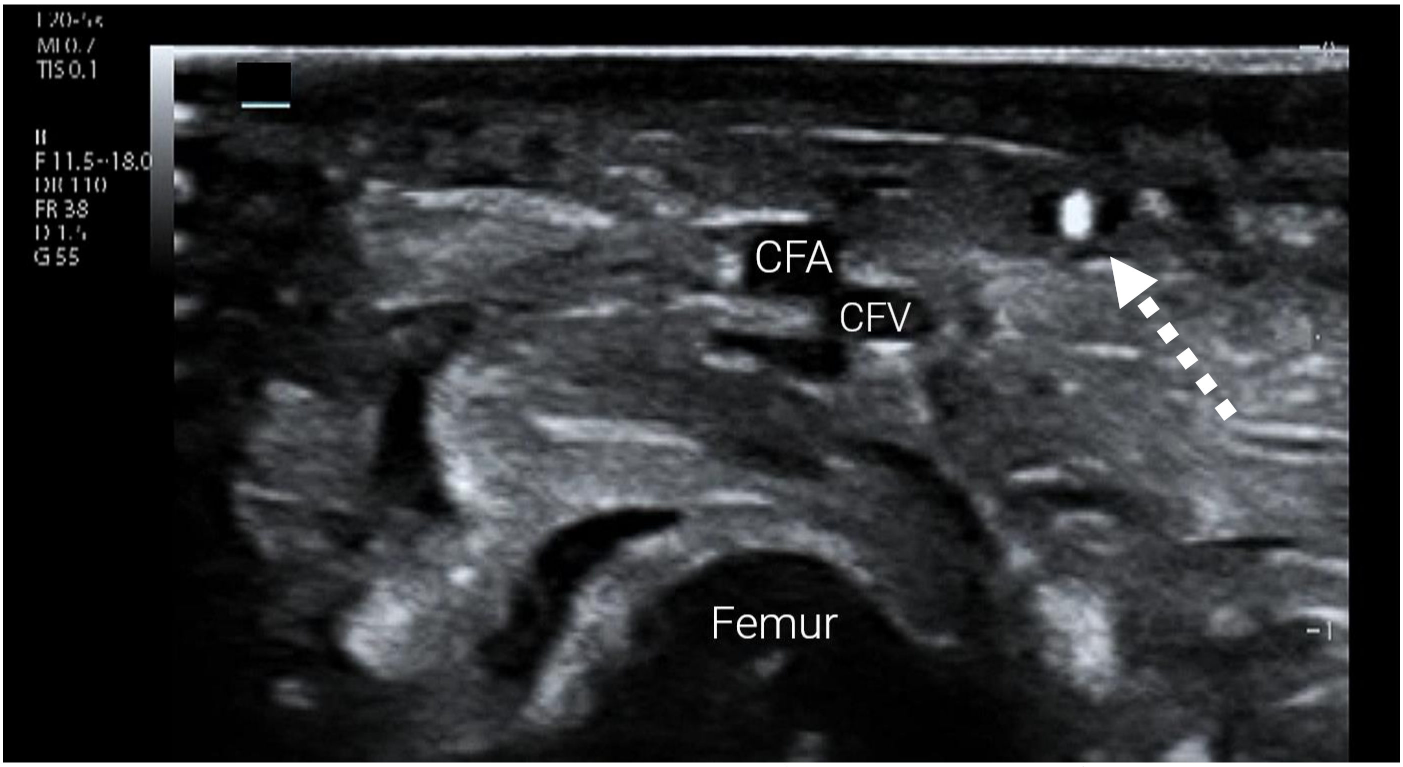

Fig.6. inguinal view, linear probe. Catheter in transverse view (white dot, dotted arrow) is visible in the sapheno-femoral junction. CFA – common femoral artery, CFV – common femoral vein. The catheter is more prominent in this view due to intraprocedural imaging with the guidewire not removed.

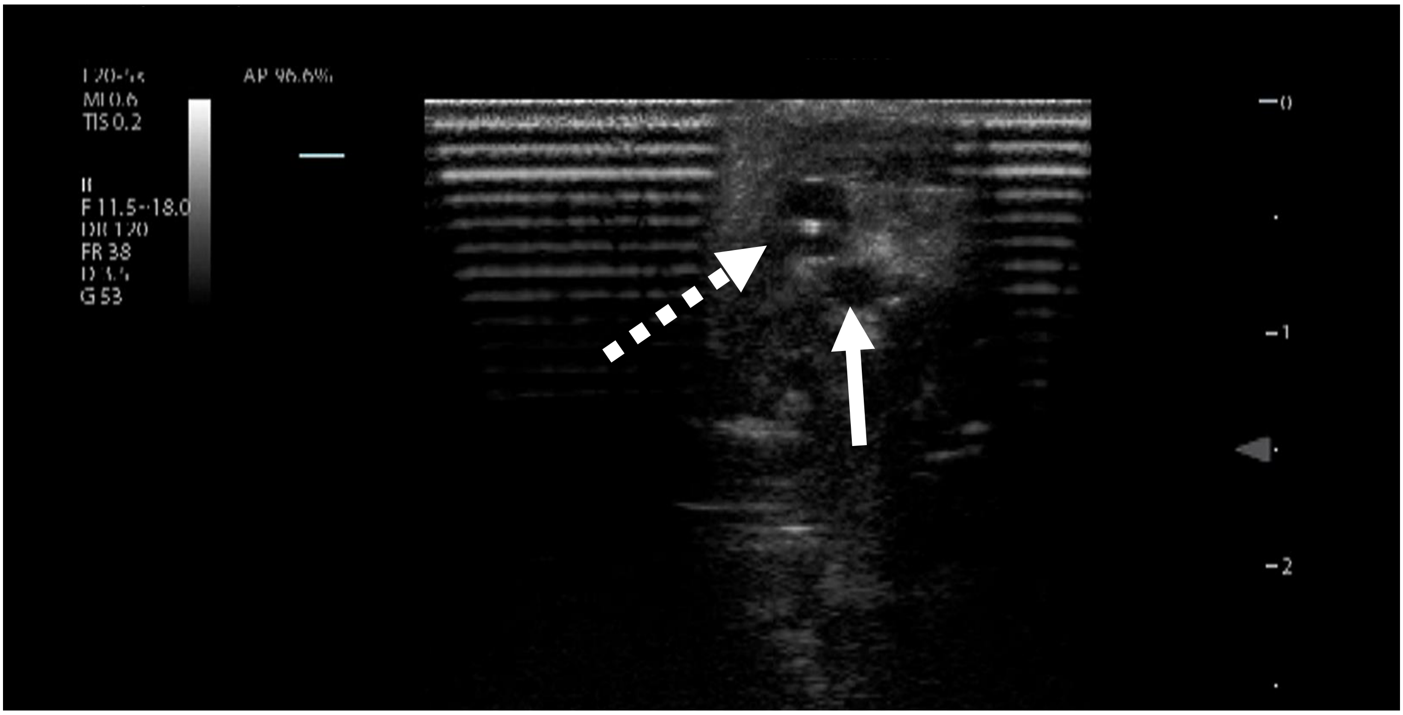

Fig.6. transverse jugular view, linear probe. Dotted arrow shows the catheter visible within the internal jugular vein. Below, on the left, internal jugular artery is also visible (white arrow).

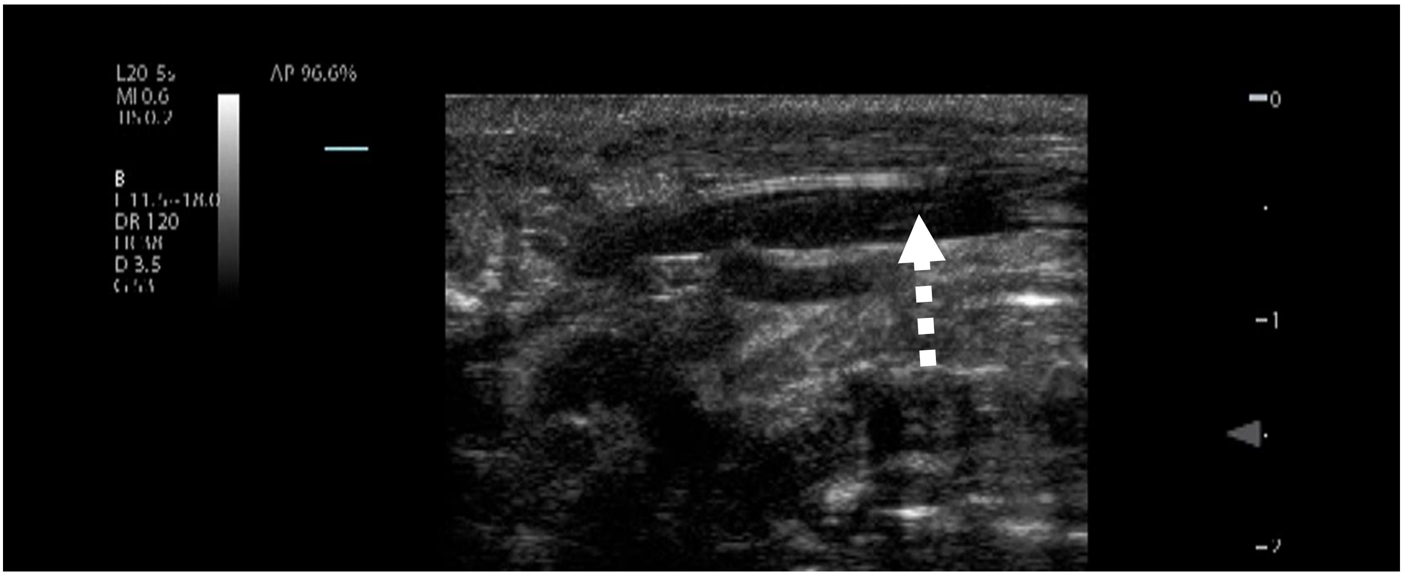

Fig.7. longitudinal jugular view, linear probe in the same patient as above. Dotted arrow shows the catheter tip clearly visible within the internal jugular vein. This PICC line was inserted form the upper limb resulting in tip malposition in the neck.

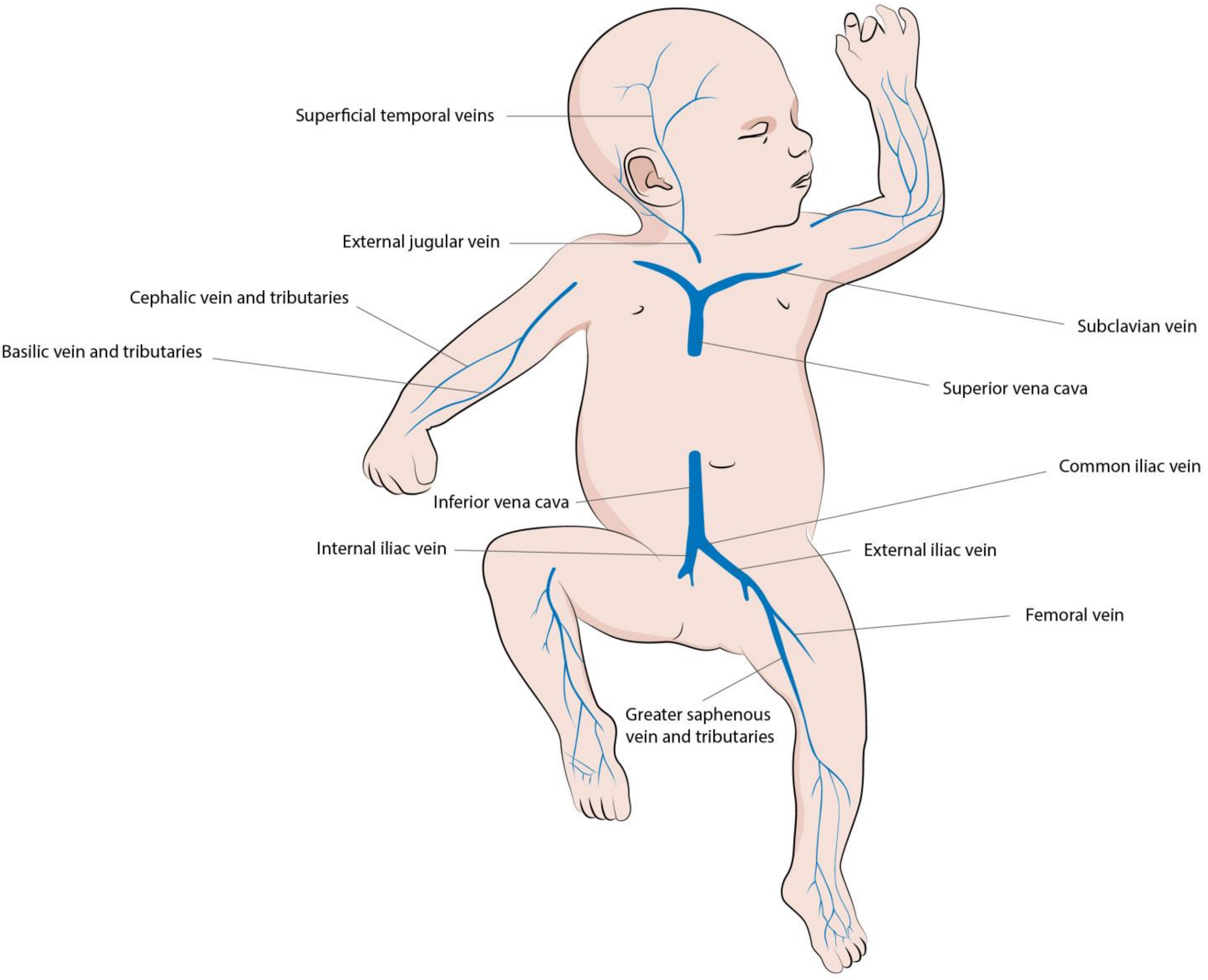

Fig. 8. General reference for central venous vessels and their tributaries used for PICC insertion.
